# Distinct stages during colonization of the mouse gastrointestinal tract by *Candida albicans*

**DOI:** 10.3389/fmicb.2015.00792

**Published:** 2015-08-05

**Authors:** Daniel Prieto, Jesús Pla

**Affiliations:** Departamento de Microbiología II, Facultad de Farmacia, Universidad Complutense de MadridMadrid, Spain

**Keywords:** commensalism, adaptation, fitness, mouse gut, *Candida albicans*

## Abstract

*Candida albicans* is a member of the human microbiota, colonizing both the vaginal and gastrointestinal tracts. This yeast is devoid of a life style outside the human body and the mechanisms underlying the adaptation to the commensal status remain to be determined. Using a model of mouse gastrointestinal colonization, we show here that *C. albicans* stably colonizes the mouse gut in about 3 days starting from a dose as low as 100 cells, reaching steady levels of around 10^7^ cells/g of stools. Using fluorescently labeled strains, we have assessed the competition between isogenic populations from different sources in cohoused animals. We show that long term (15 days) colonizing cells have increased fitness in the gut niche over those grown *in vitro* or residing in the gut for 1–3 days. Therefore, two distinct states, proliferation and adaptation, seem to exist in the adaptation of this fungus to the mouse gut, a result with potential significance in the prophylaxis and treatment of *Candida* infections.

## Introduction

Fungal agents are an important cause of nosocomial infections, which are a primary health problem in several countries (Pfaller and Diekema, 2007; Alangaden, 2011). Their treatment, especially of systemic nosocomial infections is complicated by the limited number of antifungals available (Ostrosky-Zeichner et al., 2003) and the emergence of resistance to some of the most commonly used (Cowen et al., 2015). *Candida albicans* is the most frequent pathogenic fungus found in humans and the 4th most common cause of blood borne systemic nosocomial infections. This yeast is found as a harmless commensal in the human population, residing mainly in the gastrointestinal and vaginal tract. Under impairment of physical host barriers and/or alteration of immune defenses (Romani, 2004), *C. albicans* is able to translocate through the mucosa and gain access to internal organs, causing a systemic disease and organ failure (Mavor et al., 2005). Genetic evidence supports that *C. albicans* strains found in the bloodstream are genetically similar to those found in rectal isolates and that increased gut colonization is, as maybe expected, a risk for dissemination (Miranda et al., 2009). *C. albicans* infections are, therefore, mainly endogenous (Odds et al., 2006). Understanding the mechanisms that control the establishment of this microbe in host niches is essential for designing strategies to treat and prevent fungal infections (Pierce and Lopez-Ribot, 2013; Paul and Moye-Rowley, 2014).

The development of genetic tools for this organism (De Backer et al., 2000; Berman and Sudbery, 2002; Hernday et al., 2010; Vyas et al., 2015) has enabled the identification of several virulence genes, although the concept of virulence itself may be difficult to define for commensal microbes (Casadevall and Pirofski, 2003). Most virulence genes identified to date encode adhesion molecules, metabolic enzymes, regulators of morphologic changes (such as the white-opaque) and members of signaling pathways (Mayer et al., 2013). Of particular interest is dimorphism, which is triggered in response to certain environmental factors, and that influences *C. albicans* ability to invade and proliferate within tissues and cope with immune cells (Saville et al., 2003, 2006; Gow et al., 2012; Jacobsen et al., 2012). These works have mainly used the mouse systemic model of infection and, more recently, alternative non-vertebrate models (Fuchs and Mylonakis, 2006; Arvanitis et al., 2013) that, unfortunately, do not mimic the natural route of *C. albicans* infections in humans. The development of models of gastrointestinal commensalism (Koh, 2013) has been a main advantage to analyze fungal factors influencing colonization. Although initial studies involved neonatal mice (de Repentigny et al., 1992), the most common system uses adult mice with a significant chemotherapy-driven microbiota reduction (Kinneberg et al., 1999; Mellado et al., 2000; Wiesner et al., 2001). These models have enabled the definition of the role that neutrophils have in the control of *C. albicans* dissemination (Koh et al., 2008) as well as provided experimental support for the role that certain yeast may have in the control and outcome of *C. albicans* colonization (Jawhara and Poulain, 2007). The development of these models has enabled to define the role that certain genes have on the commensal program promoting colonization via yet undefined regulatory circuits (White et al., 2007; Pierce and Kumamoto, 2012; Pande et al., 2013; Ṕerez et al., 2013; Prieto et al., 2014).

In this work we have addressed the temporal dependence of *C. albicans* adaptation to the mouse gastrointestinal tract. We have previously developed a dual labeled system that enables tracing populations in the gut via flow cytometry or standard viable colony counting (Prieto et al., 2014). Using this system and measuring competitive fitness between different fungal populations, we were able to show how *C. albicans* cells adapt to the commensal status in a timely fashion.

## Materials and methods

### Strains and growth conditions

*C. albicans* strains used in this work were CAF2-GFP (COA6-3) and CAF2-dTOM2 (PPD7), both described in a previous work (Prieto et al., 2014). The only genetic difference between the two strains is the fluorescence label (GFP or RFP), which does not impose any loss of fitness to the population allowing the differentiation of each strain from a mixed population in colonies (Prieto et al., 2014). According to our nomenclature (Table 1), Ca-n refers to *C. albicans* cells grown *in vitro* (YPD), Ca-g refers to cells present in the gut and Ca-f to cells derived from stools (in a cohousing experiment). In some experiments, the genetic label of the cells (GFP, RFP) was swapped among the different physiological status (n, g, or f) to discard any role of the fluorescent label in terms of fitness. In these cases, all data have been plotted together as they were practically identical.

**Table 1:** **Nomenclature of *C. albicans* populations studied in this work**.

**Nomenclature**	**Origin**	**State**	**Description**
Ca-n	*C. albicans* cells obtained under standard laboratory conditions	*In vitro*	Stationary overnight cells
Ca-g	*C. albicans* cells present in the gut	*In vivo*	Short term (2 days, Ca-gS) or Long term (>14 days, Ca-gL) after gavage
Ca-f	*C. albicans* cells derived from animal feces	*Ex vivo*	Ca-fS (Short term, 2 days) or Ca-fL (Long term, 21 days) refers to original state of the animal in the moment of cohousing

Ca-n population was obtained from an overnight (24 h) culture at 37°C in YPD liquid medium (2% glucose, 2% peptone, 1% yeast extract) of CAF2-GFP (COA6-3) or CAF2-dTOM2 (PPD7). These strains were obtained starting from fresh YPD plates derived from a −80°C glycerol stock and kept at 4°C for 2–4 weeks. Overnight cells were recovered by a low speed centrifugation (3000 r.p.m for 5 min) and resuspended in PBS. Different number of cells (see description of each experiment) in a volume of 100 μL were inoculated by gavage. Stools samples were plated in SD solid medium (2% glucose, 0.5% ammonium sulfate, 0.17% yeast nitrogen base and 2% agar) plus amino acids and chloramphenicol (10 μg/mL) for colony counting.

### *In vivo* procedures

The experiments involving animals performed in this work were carried out in strict accordance with the regulations in the “Real Decreto 1201/2005, BOE 252” for the Care and Use of Laboratory Animals of the “Ministerio de la Presidencia,” Spain. The protocol was approved by the Animal Experimentation Committee of the University Complutense of Madrid (Permit Number: CEA 25/2012, BIO2012-31839-1). All efforts were made to minimize suffering. Mice euthanasia was performed by CO_2_ inhalation following standard protocols (AVMA Guidelines for the Euthanasia of Animals: 2013 Edition). The number of animals used was minimized for ethical reasons.

Female mice C57BL/6 obtained from Harlan Laboratories Inc. (Italy) were used within an age of 7–10 weeks-old. Mice housing and other non-invasive procedures took place in the animal facility from the Medical School of the Universidad Complutense de Madrid. We used the protocol for studying commensal colonization that we have previously described (Prieto et al., 2014) with minor modifications. After 4 days of antibiotic pretreatment (2 mg/mL Streptomycin, 1 mg/mL Bacitracin, and 0.1 mg/mL Gentamicin) in drinking water, *C. albicans* cells were inoculated in a single gavage with a specific dose. In some experiments, a second inoculation was performed on days 2, 15, or 21 to establish a competition among different populations and *in vitro* cells. In the cohousing experiments, mice were maintained in the same cage without any artificial inoculation. For all the experiments, colonization was assessed by measuring CFUs (Colony Forming Units) of *C. albicans* in freshly obtained stools. Fresh stool samples were collected from each individual on different days, mechanically homogenized in PBS to 40 mg/mL and viable cells were determined on SD-agar plates. Colonies were associated to a specific population in accordance to the colony color (red colonies, RFP expressing cells; white-greenish colonies, GFP expressing cells). Proportions of populations lower than ~1:10,000 could not be distinguished on plates. The detection limit of our technique in competition experiments is ~2500 cells/g stools, which is plotted in some figures. When fungal levels were below this limit, colonization was considered 0 for graphical representation. To normalize fungal levels with the levels at the intervention we used competition index (CI). For a population A vs. a population B, CI was calculated in the following way: CI^A/BDayX^ = (CFU^Ca−A^/CFU^Ca−B^)_DayX_/(CFU^Ca−A^/CFU^Ca−B^)_Day1_. The value of 2500 CFU/g stools was used for groups with colonization levels below detection limit to enable CI calculation.

### Statistical analysis

All statistics have been calculated using GraphPad Prism 5 software. Linear regression was used to compare the decay of colonization of fungal populations tested after antibiotic removal on different days. To determine statistical differences among log_2_ CI values from different experiments and days, we performed One-Way ANOVA plus Bonferroni's multiple comparison tests to compare more than two groups and Student's two-tailed unpaired *t*-tests when there were just two groups. Data shown in figures are either each replicate and/or mean ± standard error.

## Results

### Equilibrium levels of *C. albicans* in gut colonization are independent of fungal dose

**Figure 1:**
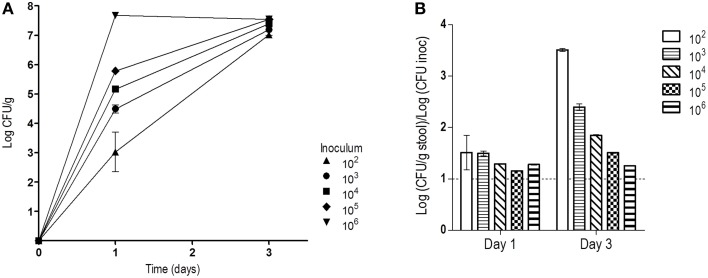
**Influence of the inoculum dose in the colonization levels**. After four days of antibiotic treatment, different doses of *C. albicans* were orally given. **(A)** Fungal loads in stools (log CFU per gram, mean ± SEM) from each group (*n* = 2) on days 1 and 3. **(B)** Relation between levels detected in stools and dose on days 1 and 3 (mean ± SEM).

Treatment of mice with oral antibiotics has been a broadly used strategy to allow high *C. albicans* colonization levels after gastrointestinal inoculation (Wiesner et al., 2001; Koh, 2013). In these protocols, a high dose of *C. albicans* is normally given either by a single gavage or via drinking water *ad libitum*. Oral doses (about 10^7^ CFUs) are in the range of the levels later obtained from stools (10^6^–10^7^ CFUs/g stool). It is normally accepted that a high inoculum may help gut establishment of *C. albicans*. We tested this assumption by inoculating 5 groups of mice (*n* = 2) with 100 μL of serial dilutions of a 10^7^ CFUs/mL solution of stationary *C. albicans* phase cells. Mice therefore received 10^6^, 10^5^, 10^4^, 10^3^, or 10^2^ *C. albicans* cells in a single dose and stool fungal levels were determined in the following days. At day 1, CFUs obtained from stools correlated well with the inoculated dose and were roughly 10x higher (in CFUs/g) than the dose. At day 3, they all reached similar high colonization levels (7.3 ± 0.23, mean of Log (CFU/g) ± SEM), independently of the dose received (Figure 1). Ratios between stool concentration values at day 1 and dose inoculum was found to be very similar for all groups, 1.3 ± 0.15 [Log (CFU/g)/Log (CFU inoculated), mean ± SEM], suggesting that cells proliferated rather similarly during this period. This result indicates that the first 72 h after inoculation are critical for *C. albicans* to proliferate and attain stable stationary colonization levels which are then properly maintained in the next days. We hypothesized that during this period cells focus on proliferation, while at later stages, *C. albicans* may rely on adaptation to the new environment.

### *C. albicans* colonization time affects fall of fungal levels after antibiotics removal

**Figure 2:**
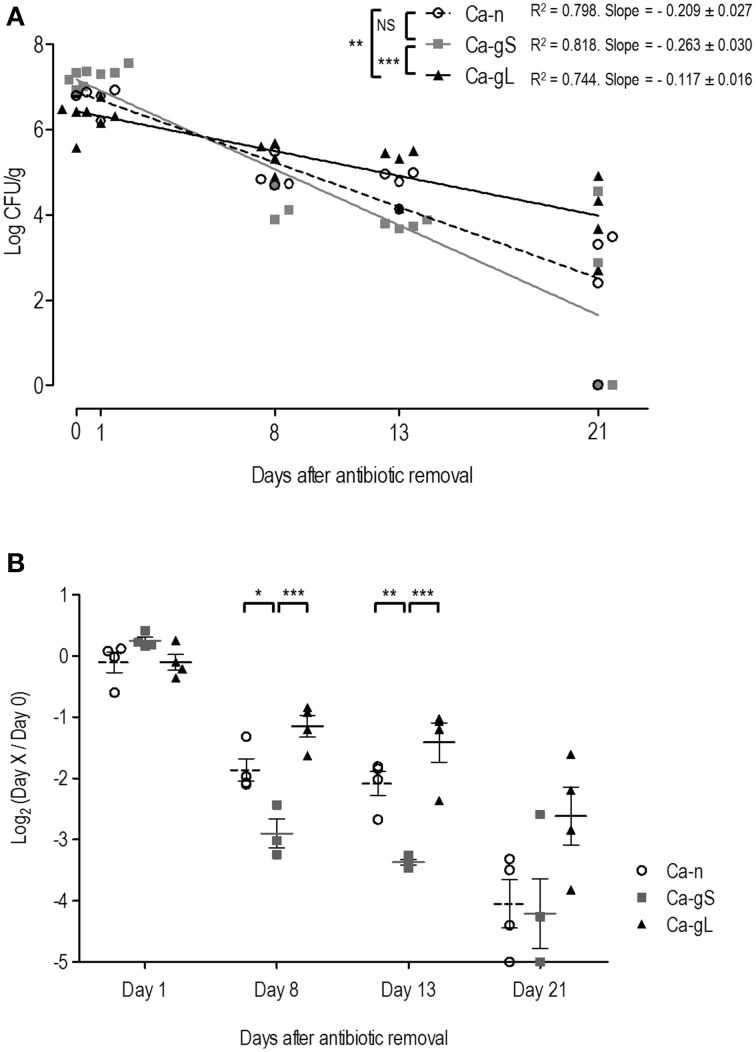
**Decay of colonization of different** ***C. albicans*** **populations after antibiotic removal**. Antibiotic treatment was replaced with sterile water in each group (*n* = 4) on day 0 (Ca-n, open circles), day 2 (Ca-gS, gray squares) or day 23 (Ca-gL, black triangles) from the inoculation of 10^7^ cells of *C. albicans*. Each symbol represents data from an individual mouse. **(A)** Evolution of fungal loads in stools (log CFU per gram) along 21 days after the antibiotic treatment was removed. **(B)** Ratios of fungal levels (CFU per gram) in relation to the day when antibiotics was removed (day 0). ^*^p < 0.05, ^**^p < 0.01, ^***^p < 0.001.

It is known that modification of the bacterial microbiota is needed to allow a stable colonization of *C. albicans* (Koh et al., 2008). Our colonization protocol is based on application of a specific antibiotic regime to the mice via the drinking water (Wiesner et al., 2001) and when removed, fungal loads decrease (Prieto et al., 2014) allowing efficient removal of *C. albicans* from the mouse gut. We wondered if this decline would occur similarly to gut-adapted or non-adapted populations of *C. albicans*. We used for this purpose cells obtained from *in vitro* standard laboratory growth conditions (that we call Ca-n, for non-adapted), and cells already present in the mouse gut (Ca-g, for gut adapted). We also distinguished between short-term adapted (Ca-gS) cells for those present in the gut for 2 days and long-term adapted (Ca-gL) when they have established in the gut for 2 or more weeks (see Table 1 and Materials and Methods). We therefore treated 3 groups of mice with antibiotics for 4 days and afterwards 10^7^ *C. albicans* cells were inoculated to each individual. Antibiotic therapy was removed from experimental groups at different time points: group 1 mice lacked antibiotics in drinking water immediately after inoculation (day 0), in group 2, antibiotic therapy was removed 2 days after inoculation (Ca-gS) while in group 3 cells were allowed to colonize for 23 days (Ca-gL). After oral antibiotics were removed, fungal loads on stools were analyzed at different days (0, 1, 8, 13, and 21). To assess the colonization decrease rate and be able to compare the behavior of different groups, we calculated linear regression of fungal loads (log CFU/g) at different time points (Figure 2A). Ca-gL group showed the lowest fall of colonization (0.117 log units per day), while groups Ca-gS and Ca-n displayed slopes of 0.263 and 0.209, respectively, roughly inversely correlating with colonization time. Actually, only Ca-gL group linear regression differed significantly from the other two groups (*p* < 0.01 for Ca-n and *p* < 0.001 for Ca-gS). Ca-gS and Ca-n did not show a significant different behavior. Since each group presents a different colonization level after antibiotic removal, we calculated ratios of fungal loads referring to the value attained at day 0 (Figure 2B). At day 1, all groups present similar log_2_ values close to 0, indicating that no important changes had affected the *C. albicans* populations yet. All groups experienced an important drop in fungal loads in the following days. This effect was less pronounced in Ca-gL group and, interestingly, much more in Ca-gS population. The latter was significantly different from the other two groups, on both days 8 and 13. However, on day 21 no significant differences among groups were observed.

### Long-term (but not short-term) gut-adapted *C. albicans* cells displace non-adapted fungal populations

**Figure 3:**
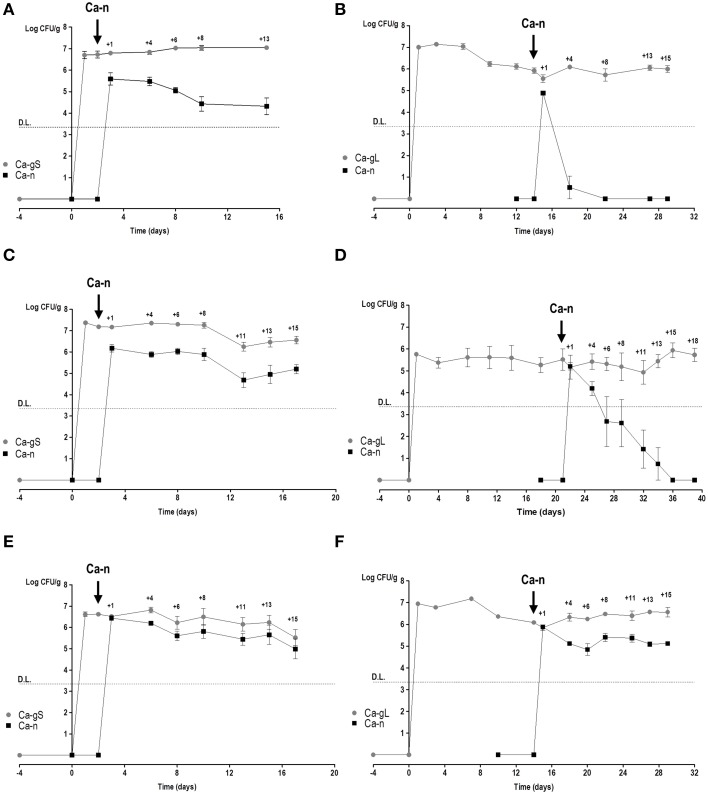
**Competition in colonization among Ca-n and Ca-g** ***C. albicans*** **populations**. Competition between Ca-n and Ca-g populations was performed through inoculation of cells from *in vitro* culture in the day and doses indicated. Colonies were associated to a specific population in accordance to the fluorescent label. A value of 0 is attributed to non-detected-populations. Stool levels (log CFU per gram, mean ± SEM) are represented along the time. **(A)** Ca-gS vs. 10^6^ cells of Ca-n (*n* = 3); **(B)** Ca-gL vs. 10^6^ cells of Ca-n (*n* = 7); **(C)** Ca-gS vs. 5 × 10^6^ cells of Ca-n (*n* = 5); **(D)** Ca-gL vs. 5 × 10^6^ cells of Ca-n (*n* = 5); **(E)** Ca-gS vs. 10^7^ cells of Ca-n (*n* = 6); and **(F)** Ca-gL vs. 10^7^ cells of Ca-n (*n* = 4). DL refers to detection limit as explained in Materials and Methods section.

In order to determine the fitness of different adapted *C. albicans* cells, we used our recently developed red or green fluorescent gene reporter system (Prieto et al., 2014) to distinguish between these populations. We used cells labeled with either GFP or RFP which were allowed to colonize mice thus generating Ca-gS and Ca-gL populations. Mice were inoculated by gavage with *C. albicans* cells (normally GFP-labeled) and after 2 or 15–21 days, a new Ca-n population was introduced (then RFP-labeled) to allow competition with already established Ca-gS and Ca-gL (respectively) present populations. At different times, the abundance of each population was determined in stool samples from every mouse (Figure 3). Different doses of Ca-n were tested to ensure that competition would not be critically dependent on the load (dose) of *C. albicans* inoculated.

We noticed that a Ca-n population easily maintains sustained levels of colonization in the gut when it competes with Ca-gS (Figures 3A,C,E). Although lower levels of Ca-n are detected in stools when inoculating low doses (Figures 3A,C), the pattern from the first day is rather constant. However, in the presence of a Ca-gL population, Ca-n is unable to establish and attain high colonization loads (Figures 3B,D,F). Only upon inoculation of a high dose (10^7^ cells) of Ca-n cells, these cells were eventually able to establish in the gut. Notably, the colonization levels attained by Ca-n are lower to those found at day 1, which probably reflect transient passage through the gastrointestinal tract but not equilibrium levels (Figure 3F).

**Figure 4:**
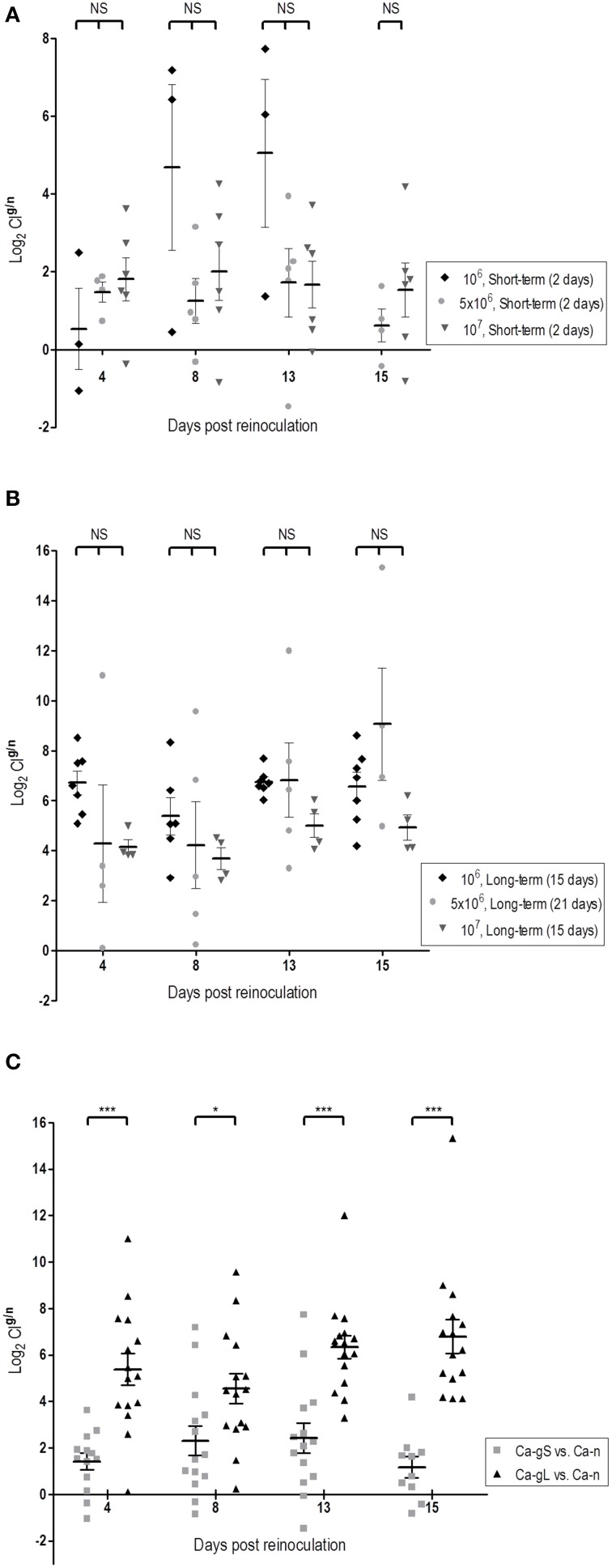
**Dose and time influence in the colonization fitness**. Differences in fitness are determined through comparison of CI values (log_2_). Each symbol represents data from an individual mouse. **(A)** Effect of dose among Ca-gS groups (*n* = 3–6). **(B)** Effect of dose among Ca-gL groups (*n* = 4–7). **(C)** Effect of the time past in the gut regardless the dose (*n* = 14–16). ^*^p < 0.05, ^***^p < 0.001.

Since each strain is inoculated on different days (days 2, 15, or 21), it is difficult to compare the evolution of the populations in competitive fitness. We chose to use the proportions of strains determined in stool samples at day 1 as this value nicely correlates with the proportion in the mix inoculated in standard colonization competition assays in mice (Figure S1). We used colonization competition index (CI) (see Materials and Methods) as it actually reflects the ability of one population to persist over the other one. Using this parameter, we have analyzed whether the dose and/or stage of previous population influence the subsequent competition. While a high *C. albicans* dose (10^7^ cells) helps Ca-n to reach higher fungal levels on day 1, it does not really impact the outcome of the evolution of the population levels as no statistically significant differences were detected in the CI^*g*/*n*^ ratio at any dose administered, neither in short-term or long-term populations (Figures 4A,B). However, strikingly clear differences appear when comparing Ca-gS and Ca-gL groups, regardless the dose: the CI^*g*/*n*^ was always higher for *C. albicans* cells that were in the gut for a long time, that is Ca-gL outcompetes more efficiently the new Ca-n population than Ca-gS (Figure 4C). Moreover, we only find values for CI^*g*/*n*^ below 1 (negative as a logarithm) in the Ca-gS group. Remarkably, the mean of this index does not significantly changes within a group of mice over time (Figure 4C) with log_2_ values of 1.41 ± 0.36 (day 4), 2.31 ± 0.64 (day 8), 2.41 ± 0.64 (day 13), and 1.17 ± 0.45 (day 15) for short-term group; and 5.38 ± 0.69 (day 4), 4.54 ± 0.64 (day 8), 6.34 ± 0.49 (day 13), and 6.79 ± 0.73 (day 15) for Ca-gL group (log_2_CI^*g*/*n*^, mean ± SEM).

Collectively, these results demonstrate that Ca-gL cells show increased fitness over Ca-gS in competition experiments with a freshly inoculated *C. albicans* cell population, therefore reflecting different adaptation to the host.

### Gut-adapted *C. albicans* cells are efficiently maintained during cohousing experiments

In order to determine more precisely the behavior of gut-adapted populations, we performed competition experiments between different gut adapted *C. albicans* populations. For this purpose, we considered another type of adapted population, Ca-f (from feces-derived *C. albicans*, Table 1). We first determined the ability of antibiotic-treated mice to become colonized from stool samples; this possibility has been recently shown to occur and provide experimental evidence for horizontal transmission under specific experimental conditions (Cutler et al., 2011). We cohoused (same cage) two control mice (i.e., non-inoculated with *C. albicans*, only receiving PBS) with 8 mice inoculated with *C. albicans* (Figure S2). PBS-mice reached high fungal loads in stools as early as one day after the cohabitation (mean = 5.79, logCFU/g), although still not reaching the levels of *C. albicans*-mice (mean = 7, logCFU/g). At day 2, comparable fungal loads were found in both groups, PBS or *C. albicans*-inoculated (mean = 7.12 and 7.28, respectively). We therefore conclude that cohousing mice is an efficient and natural way to spread out *C. albicans* gut-adapted populations in mice under our conditions.

**Figure 5:**
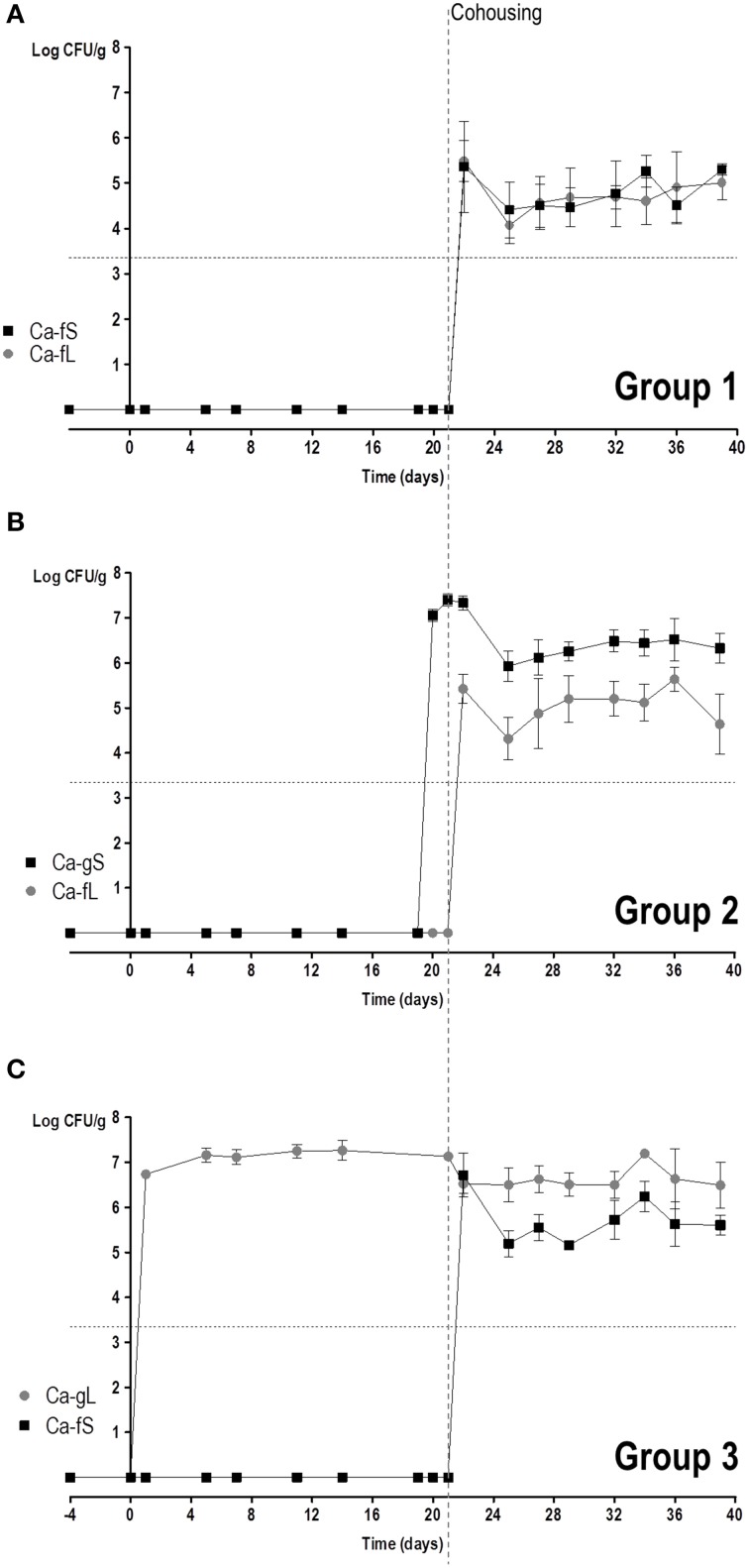
**Competition in colonization among Ca-g and Ca-f** ***C. albicans*** **populations**. Competition between Ca-g and Ca-f populations was performed through putting together mice non-colonized (group 1) and mice already colonized for 2 or 23 days (groups 2 and 3, respectively). Colonies were associated to a specific population in accordance to the fluorescent label. Each symbol represents data from an individual mouse. Stool levels (log CFU per gram) are represented along the time. **(A)** Group 1: Ca-fS vs. Ca-fL (*n* = 3); **(B)** Group 2: Ca-gS vs. Ca-fL (*n* = 3); **(C)** Group 3: Ca-gL vs. Ca-fS (*n* = 3).

**Figure 6:**
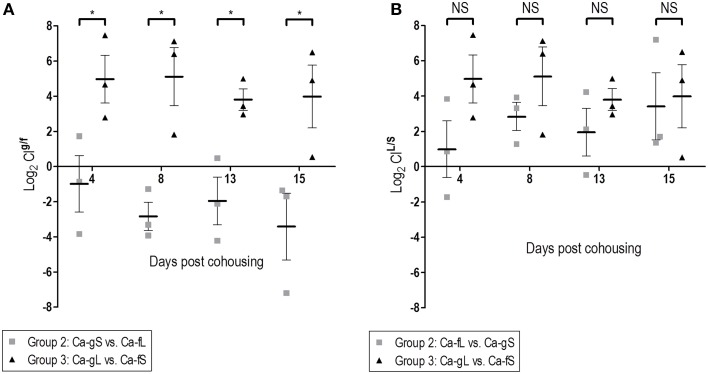
**Dose and time influence in the colonization fitness**. Differences in fitness are determined through comparison of CI values (log_2_). **(A)** Effect of the time past in the gut. **(B)** Effect of the source. ^*^p < 0.05.

We then studied the behavior of short (Ca-fS) and long term (Ca-fL) Ca-f populations in competition with different Ca-g populations. For this purpose, 3 groups of mice were allowed to cohabit (same cage) together: Group 1 had no fungi and just received antibiotic treatment; Group 2 had colonizing *C. albicans* CAF2-dTOM2 on day 2 after gavage (Ca-gS) while Group 3 had *C. albicans* CAF2-GFP on day 21 (Ca-gL) after gavage (see Figure S3). The appearance of *C. albicans* cells from different origins was followed in the stools of the animal in the next 3 weeks. Given the above results, the source of exogenous (non-gavage mediated) colonization is presumed to be the stool population (either Ca-fS or Ca-fL). As shown in Figure 5, all mice presented both GFP and dTOM2-labeled strains in their stools. This result indicates that Ca-fS and Ca-fL are efficient to become established within mouse gut even in the presence of a Ca-g population. However, each population showed a different evolution. In group 1, as animals were devoid of fungi, competition is initially established between Ca-fS and Ca-fL. Both GFP and dTOM2 labeled cells were able to maintain constant levels (≈10^5^ yeasts per gram, Figure 5A), indicating that a *C. albicans*-free gut niche is not restrictive enough to discriminate between those two populations. Identical levels achieved by each strain in the first day indicate that both populations were similarly accessible to mice. In group 2 mice, we also found ≈10^5^ CFUs/g stools of Ca-fL, while the previous endogenous population (Ca-gS) showed colonization over 10^6^ CFUs/g. For group 3, steady state levels for Ca-fS and Ca-gL populations were around 10^6^ and 10^7^ yeasts per gram, respectively. Interestingly, Ca-fS population peaked at day 1 showing values very similar to endogenous population (mean = 6.7 vs. 6.5, logCFU/g) and later showing a slight decrease. Group 2 involves competition between Ca-gS and Ca-fL (Figure 5B). The latter did not achieve really high colonization levels at day 1 (Figure 5B). However, it is evident that it is favored in competition since mean log_2_ CI^*g*/*f*^ values were negative (Figure 6A), that is −0.99 ± 1.61 (day 4), −2.84 ± 0.80 (day 8), −1.95± 1.36 (day 13) and −3.42 ± 1.89 (day 15) (mean ± SEM). In group 3, Ca-fS is more efficient in immediate survival as it reached the highest loads at day 1, nevertheless it doesn't maintain this level during the rest of the experiment (Figure 5C). In contrast with the previous group, log_2_ CI^*g*/*f*^ values were all positive with means around 4–5, confirming that Ca-fS population fails to outcompete Ca-gL. Taken together, this reveals that the population Ca-fL suffers a slight impairment in the early colonization stage (establishment and immediate survival) for which Ca-fS population is better adapted.

Regarding the second stage, Ca-fL population is conveniently prepared for persisting in the gut, while Ca-fS shows some difficulties competing with a population adapted to this stage (Figures 5B,C). In addition, calculated CI^L/S^ show a similar competition between long-term and short-term, independently of the adapted-*C. albicans* source (Figure 6B). Although Group 3 presented always higher CI^L/S^ means than group 2 (especially on day 4), the differences are not significant (for day 4, *p* = 0.131). However, we observe a reduction in fitness in short-term adapted populations (from either gut or feces) at all times compared with Ca-fL or Ca-gL, confirming an important difference between those populations and an advantage for long-term adaptation in persisting in the gut (Figure 6A).

## Discussion

Adaptation to host environment is a necessary trait for any commensal microbe. *C. albicans* is a member of the human microbiota and during the past few years, several efforts have been directed toward the identification of mechanisms responsible for its relationship with humans. The development of commensalism models, normally based on antibiotic-mediated microbiota reduction, is enabling the exploration of this essential aspect of the host-fungus interaction. Initial studies by Kinneberg et al. (1999), showed that inoculation with 10^3^ or 10^7^ *C. albicans* cells resulted in high colonization of the cecum after 3 or 7 days, but no information was provided for earlier time points. We show here that as early as 3 days after inoculation, steady levels of 10^7^ CFUs per gram in stools are usually attained starting from as low as 100 cells inoculated. Given the correlation between oral doses and stool levels and the increase in up to 5 logarithmic units, it seems that during this period *C. albicans* cells focus on proliferation. Since after 3 days we already observe steady colonization levels (~10^7^ CFU/g) we chose 2 days of colonization as representative of this period for comparative studies. We show here that populations that had colonized the intestine for a long time (15–21 days) (Ca-gL) display enhanced fitness over those that had been in the gut for few (2) days (Ca-gS) or *in vitro* cells (Ca-n). This is evidenced by their reduced kinetics of disappearance from the gut after removal of antibiotic therapy and by the increased competition *in vitro* cells compared with Ca-gS. In fact, a conclusion from our studies is that colonization levels do not correlate with fitness as Ca-n rapidly aquire high colonization levels but do still show decreased fitness. Therefore, we propose that initial stages of colonization in our experimental system are mainly responsible for attaining high fungal doses (replication period) but full adaptation (at least, in terms of competitiveness) is only achieved after a more prolonged time (adaptation). Similar behaviors are also observed when the adapted populations came from feces (through coprophagy). Using transcriptomal analysis, several genes have been shown to be differentially expressed in the gut (Rosenbach et al., 2010). Many of these genes were also expressed either during growth in exponential phase or post-exponential-phase, suggesting correlation with both its ability to grow quickly (characteristic of exponential phase) and the resistance to different types of stress (more prone to stationary phase) in accordance with the challenging environment (nutrient competition, pH, oxygen availability and microbial interactions) that cells encounter in this niche. Such a scenario is in accordance with recent data that indicate that identified transcription regulators of commensalism mainly affect functions involved in carbon and nitrogen metabolism (Ṕerez et al., 2013). In any case, in Rosenbach's study cells were harvested from cecum just 3 days after inoculation, so we would associate it with our Ca-gS population.

The existence of different stages in fungal colonization would, in addition, explain some observations from literature. For example, while MAPK signaling pathways are important in mouse gut colonization (Prieto et al., 2014), defects in Hog1 results in an immediate impairment to colonize mouse gut (especially when a competition assay with a wild type strain is performed), although others such as Mkc1 and Cek1 changes result in long terms defects. Moreover, it has been reported that *efh1* and *efg1* mutant cells develop higher gut establishment levels than the wild type strain and it is considered as colonization specific transcription factor (White et al., 2007; Pande et al., 2013; Pierce et al., 2013). This phenotype resembles what we observe in in the Ca-gS population, suggesting that these factors (and possibly Wor1) could play a role in this initial adaptation.

The existence of different stages for colonizing the gut has been proposed also for other microorganisms. For example, in *Vibrio cholerae* (Lee et al., 1999; Schild et al., 2007) an early period is essential for survival and multiplication *in vivo*, while prolonged period allows bacteria to live outside the host, where nutrients amounts are much lower. Host-induced shedding seems to be also a strategy used by the microbe for dissemination (Merrell et al., 2002; Almagro-Moreno et al., 2015). As *C. albicans* is a long-term permanent colonizer of the gut tract with no significant life outside the host (“after-hours”), our observations could reflect adaptation to host from domesticated laboratory strains. It has been reported that another gut-colonizer, *Salmonella typhimurium*, would derivate into two phenotypically different populations, one becoming pathogenic to improve the chances of the second one to colonize and outcompete the microbiota present in the gut (Stecher et al., 2007). In addition, different *C. albicans* levels in the gut have been proposed to be associated with the existence of hypothetical phenotypic variants (similarly than those from *S. typhimurium)* that appear during host colonization (Kumamoto, 2011). Such a scenario is in accordance with high fungal loads of *C. albicans* in the gut being a risk factor for developing a systemic infection and frequently associated with inflammatory bowel disease (Gerard et al., 2015). This interplay is multifactorial, as commensalism is not only a microbe-driven process but dependent on the immunological status of the host (Romani, 2004; Zelante et al., 2012) and even in immune competent mice, host factors (such as the mucins) can suppress virulence attributes (Kavanaugh et al., 2014).

In conclusion, we present here evidence that in *C. albicans* two different (at least, in terms of fitness) stages exist during the colonization of mouse gut with laboratory maintained strains of *C. albicans*. As this species is a non-habitual member of the mouse gut microbiota (Iliev et al., 2012), caution must be taken in interpreting these observations, but determining whether this occurs in its natural reservoir (humans) is crucial given its potential implications in the management of *C. albicans* infections.

## Funding

Work in our laboratory is supported by Grant BIO2012-31830 from Ministerio de Econom’ia y Competitividad, Grant S2010-BMD2414 from Comunidad Aut’onoma de Madrid and Grant PCIN-2014-052 from Ministerio de Economía y Competitividad (INFECT-ERANET).

### Conflict of interest statement

The authors declare that the research was conducted in the absence of any commercial or financial relationships that could be construed as a potential conflict of interest.

## Supplementary material

Figure S1 **Correlation between strains composition in the inoculum and day 1 stool population**. Data from several competition experiments were collected. Each point correspond to one experiment that include some mice (*n* = 3–6). Percentage of RFP labeled strain in the mixed inoculum and percentage of the same strain in the stools samples on day 1 were plotted and lineal regression was performed.

Figure S2 **Fungal loads in mice stools of naturally acquired *C. albicans* populations**. Eight mice out of ten were inoculated with *C. albicans* (10^7^ cells) after four days of antibiotic treatment. The other two mice were inoculated with PBS and all of them were kept in the same cage. Fungal loads in stools (log CFU per gram, mean ± SEM) from each group are represented along the time.

Figure S3 **Scheme of cohousing experiment**.
