# Supplementary figures and images for: Distinct stages during colonization of the mouse gastrointestinal tract by Candida albicans
Source: Front Microbiol. 2015 Aug 5;6:792. doi: 10.3389/fmicb.2015.00792 (PMC4525673; doi:10.3389/fmicb.2015.00792)

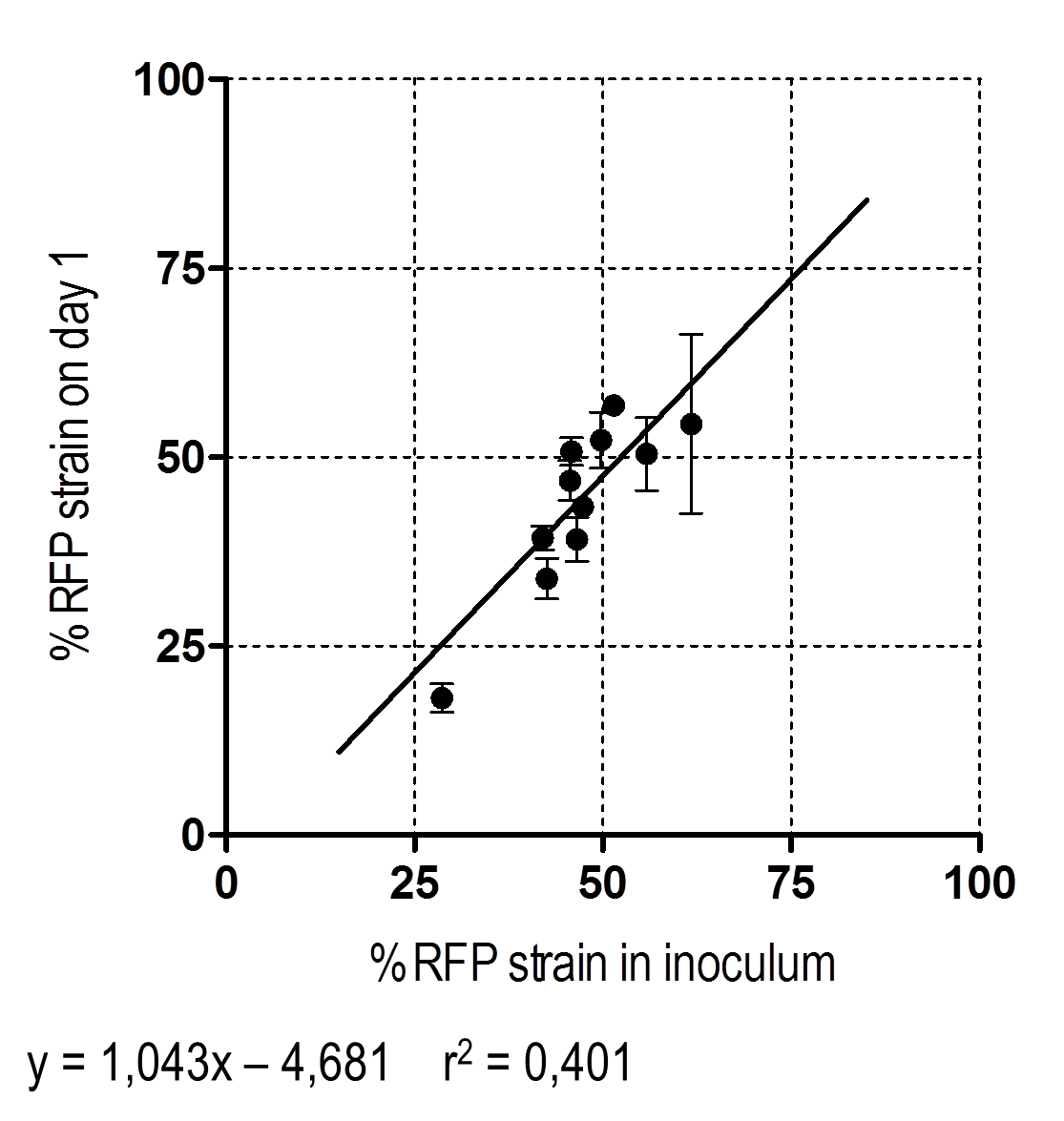

Supplement: Figure S1 — Correlation between strains composition in the inoculum and day 1 stool population. Data from several competition experiments were collected. Each point correspond to one experiment that include some mice (n = 3–6). Percentage of RFP labeled strain in the mixed inoculum and percentage of the same strain in the stools samples on day 1 were plotted and lineal regression was performed. [file Image1.TIF]

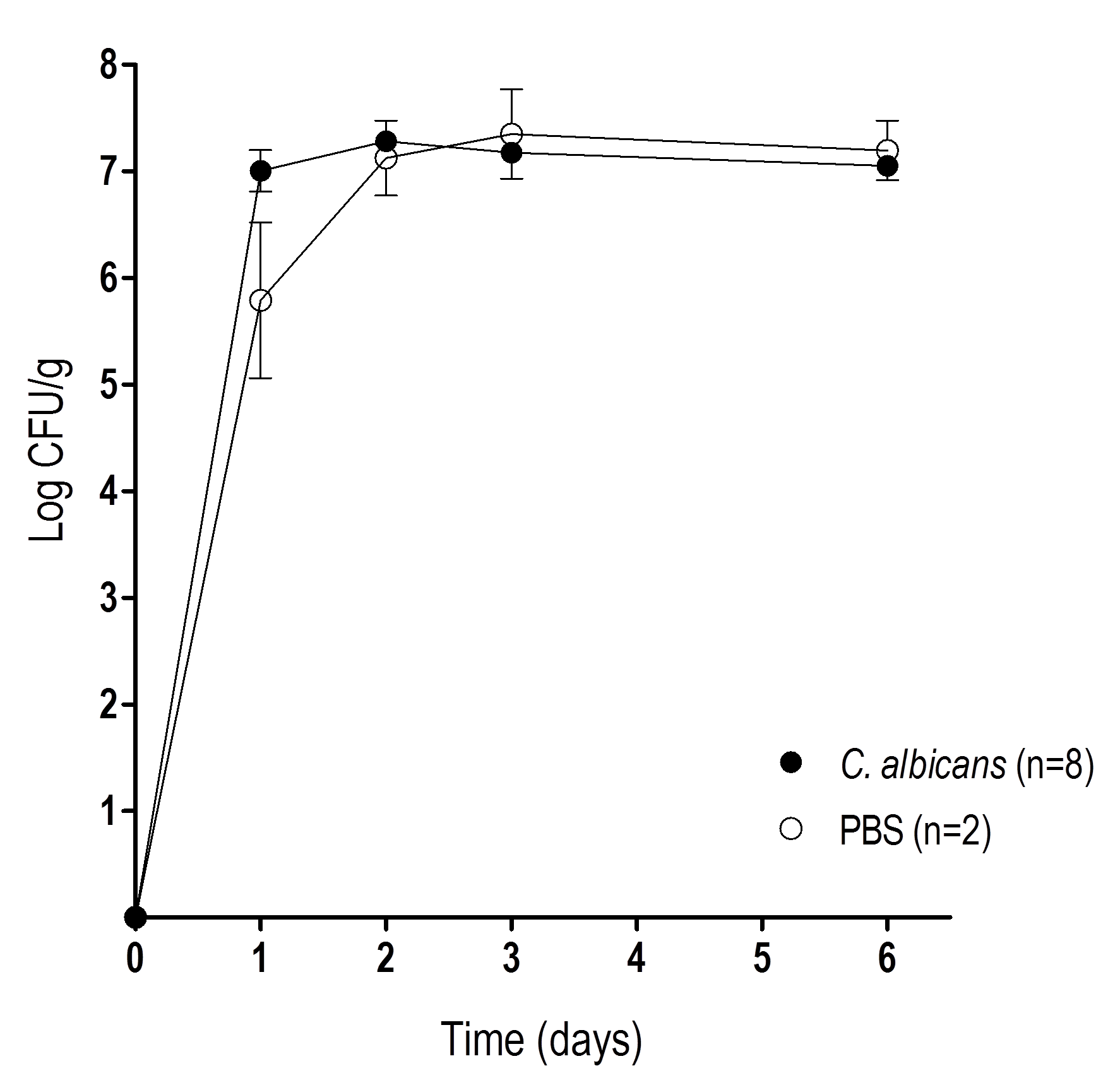

Supplement: Figure S2 — Fungal loads in mice stools of naturally acquired C. albicans populations. Eight mice out of ten were inoculated with C. albicans (107 cells) after four days of antibiotic treatment. The other two mice were inoculated with PBS and all of them were kept in the same cage. Fungal loads in stools (log CFU per gram, mean ± SEM) from each group are represented along the time. [file Image2.TIF]

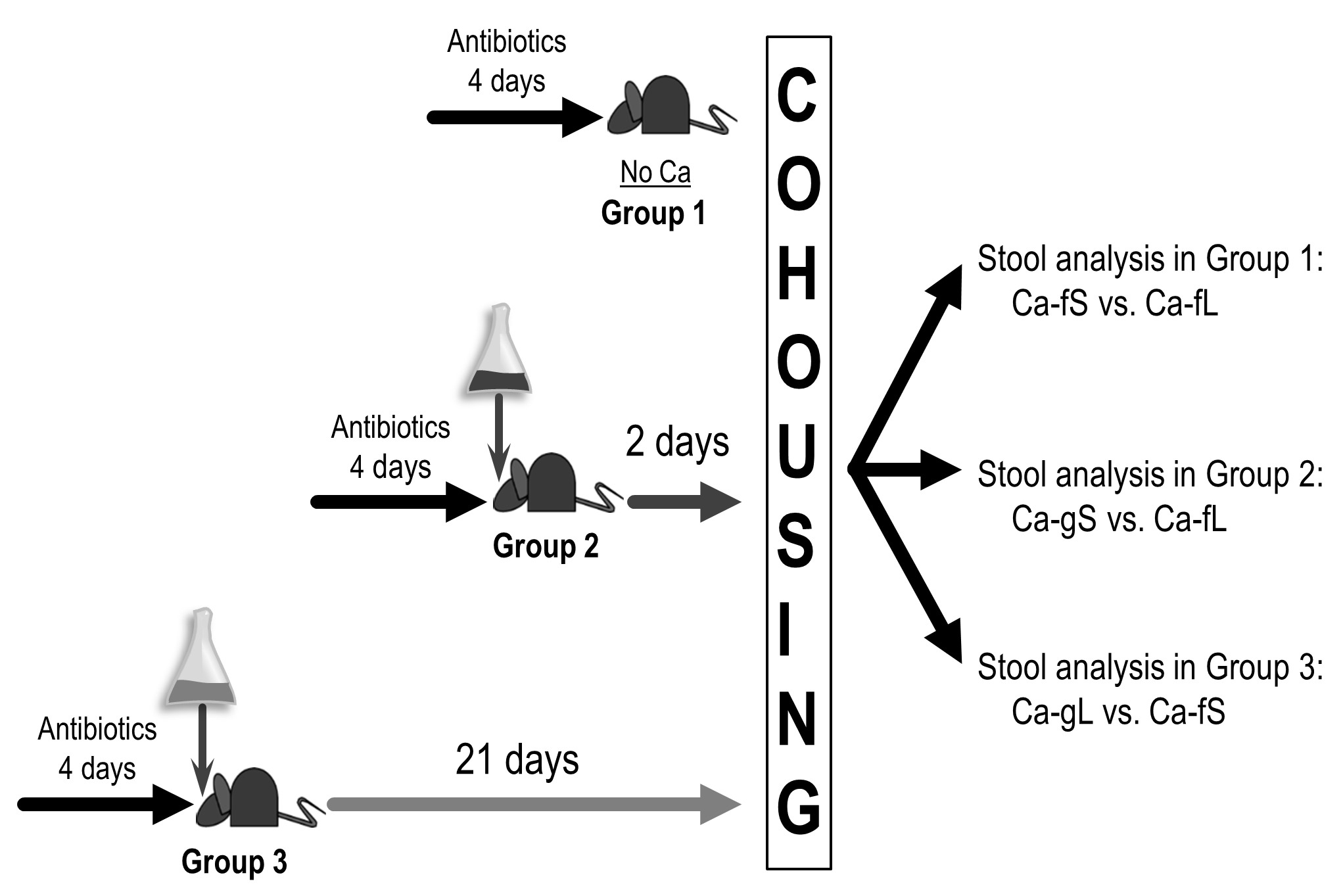

Supplement: Figure S3 — Scheme of cohousing experiment. [file Image3.TIF]
